# REalist Synthesis Of non-pharmacologicaL interVEntions for antipsychotic-induced weight gain (RESOLVE) in people living with severe mental illness (SMI)

**DOI:** 10.1186/s13643-022-01912-9

**Published:** 2022-03-09

**Authors:** Ian D. Maidment, Geoffrey Wong, Claire Duddy, Rachel Upthegrove, Sheri Oduola, Dan Robotham, Suzanne Higgs, Amy Ahern, Gurkiran Birdi

**Affiliations:** 1 College of Health and Life Sciences, Aston University, Birmingham, B4 7ET UK; 2 Nuffield Department of Primary Care Health Sciences, University of Oxford, Oxford, OX2 6GG UK; 3 School of Psychology, University of Birmingham, Edgbaston, Birmingham, B15 2TT UK; 4 School of Health Sciences, University of East Anglia, Norwich Research Park, Norwich, NR4 7TJ UK; 5 The McPin Foundation, 7-14 Great Dover St, London, SE1 4YR UK; 6 MRC Epidemiology Unit, University of Cambridge School of Clinical Medicine, Cambridge Biomedical Campus, Cambridge, CB2 0QQ UK

**Keywords:** Antipsychotics, Weight gain, Severe mental illness, Realist research

## Abstract

**Background:**

People with severe mental illnesses (SMI) such as schizophrenia die on average 15 to 20 years earlier than everyone else. Two thirds of these deaths are from preventable physical illnesses such as hypertension, cardiovascular disease, and diabetes, which are worsened by weight gain. Antipsychotics are associated with significant weight gain. In REalist Synthesis Of non-pharmacologicaL interVEntions (RESOLVE), a realist synthesis, combining primary and secondary data, will be used to understand and explain how, why, for whom, and in what contexts non-pharmacological interventions can help service users to manage antipsychotic-induced weight gain.

**Methods:**

A five-step approach will be used to develop guidance:

1. Developing the initial programme theory

An initial (candidate) programme theory, which sets out how and why outcomes occur within an intervention, will be developed.

2. Developing the search

The initial programme theory will be refined using academic and grey literature. The proposed initial sampling frame are as follows:

Context: people living with SMI, taking antipsychotics, different types of SMI.

Intervention: non-pharmacological interventions.

Mechanisms: triggered by the intervention.

Outcomes e.g. weight, metabolic adverse events, quality of life, adherence, burden, economic.

Searching for relevant documents will continue until sufficient data is found to conclude that the refined programme theory is coherent and plausible. Lived experience (service users) and stakeholder (practitioners) groups will provide feedback.

3. Selection, appraisal and data extraction

Documents will be screened against inclusion and exclusion criteria. The text extracted from these documents will be coded as contexts, mechanisms and their relationships to outcomes.

4. Primary data collection

Realist interviews with up to 30 service users and informal carers, and 20 practitioners will gather data to support, refute or refine the programme theory.

5. Data analysis

A realist logic of analysis will be used to develop and refine the programme theory from secondary and primary data. The analysis will aim to identify practical intervention strategies to change contexts so that key mechanisms are triggered to produce desired outcomes. Guidance will be produced based on these strategies.

**Discussion:**

This realist synthesis aims to develop guidance for service users and practitioners on the most appropriate interventional strategies to manage and limit antipsychotic weight gain.

**Systematic review registration:**

PROSPERO: CRD42021268697

## Background

Antipsychotics are widely used in the treatment of schizophrenia and other severe mental illnesses (SMI). Over the last 20 years, older drugs such as haloperidol (the first-generation antipsychotics) have been replaced by the newer second-generation antipsychotics (e.g. olanzapine). The second-generation antipsychotics have fewer extrapyramidal (movement) side effects, but can cause significant weight gain, and diabetes is common [1]. Clozapine, which is uniquely effective, with no alternative treatment, is one of the worst culprits [2].

There are over 220,000 people being treated for schizophrenia in the UK at any one point in time [3]. Up to 80% of people with schizophrenia or bipolar disorder are overweight or obese [4]. One of the main causes is the side-effects of antipsychotics, with weight gains of up to 33.4 kg reported [5]. This weight gain has devastating consequences: life expectancy is reduced by 20 years in people with schizophrenia, partly related to the consequences of this weight gain [6]. A high body mass index (BMI >40) is also a risk factor for poor COVID-19 outcomes, and schizophrenia itself is a significant risk factor for mortality with COVID, even after controlling for BMI and co-morbidities such as diabetes [7].

The mental and physical health of people with SMI is a priority in the NHS long-term plan [8]. The NHS Mental Health Implementation Plan identified the need for new models of care to address the physical health needs of people with SMI [9]. Many different interventions have been tried to limit antipsychotic-induced weight gain, but there is not a clear picture of what works, for whom and in what circumstances. For example, the recent STEPWISE trial of structured lifestyle changes was unable to establish either clinical or cost-effectiveness due the complexity of the area and the need for an individualised approach [10, 11]. The benefits (or not) to patients are likely to depend on contextual influences and as an approach to evaluation, randomised controlled trials (RCTs) are not well suited to developing an in-depth understanding of these.

In RESOLVE, realist synthesis, combining primary and secondary data collection, will be used to understand and explain how, why, for whom, and in what contexts non-pharmacological interventions help service users to manage antipsychotic-induced weight gain. This approach is based on that successfully used to understand medication management in older people on complex regimens [12, 13].

Much work in this area is likely to be service evaluation type data or reports which may not be included in standard systematic reviews, but can be included in RESOLVE [14–16]. The interview component of the study will generate qualitative data on the lived experience of weight gain and what works to help manage weight from a diverse group of service users and practitioners.

## Methods/design

### Aim

The aim of the study is to use realist synthesis, including primary data collection, to develop a framework and guidance for non-pharmacological interventions to manage antipsychotic-induced weight gain in people with severe mental illness (SMI).

RESOLVE had three key objectives. First, to understand how and why non-pharmacological interventions to manage antipsychotic-induced weight gain work (or not) for particular groups of people with SMI, in certain circumstances, including in ethnic minority and other marginalised groups; second, to synthesise the findings from objective 1 into a realist programme theory for interventions to manage antipsychotic-induced weight gain in people with SMI; and finally, to use the realist programme theory developed from objective 2 to inform the development of guidance on managing antipsychotic-induced weight gain and a framework for intervention design.

Both secondary (literature review strand) and primary (interview strand) data will be used to develop and test (confirm, refute or refine) the programme theory [12, 13, 17]. A realist logic of analysis will be used to analyse and synthesise the data both within and across the two strands of this project. Stakeholder engagement will be integral throughout the realist synthesis and supported by a lived experience group (LEG) containing service users and informal (family or friends) carers, and a stakeholder group (SG) containing practitioners.

The secondary data employed will include both published research studies and ‘grey’ literature, such as policy documents and service evaluation reports. Interviews will enable the direct involvement of a purposefully selected diverse group of service users and their carers, so that people with lived experience are directly involved in refining the programme theory on which the guidance we develop will be based.

A five-step approach based on methods successfully used by the lead applicant in MEMORABLE will deliver the research [12]. Below for clarity the steps are shown as discrete entities. However, operationally, we will move seamlessly between steps as the project progresses and data is iteratively gathered and synthesised.

### Developing the initial programme theory

An initial (candidate) programme theory, which sets out how and why outcomes occur within an intervention [18] will be developed based on the collective experience of the project team with input from the LEG and SG. The initial programme theory will be mapped out as a series of steps required to reach the final desired outcome, identifying intermediate outcomes that take place either sequentially or in parallel. As the project develops, for each step, the relevant and associated context and mechanism for each outcome will be developed from data identified within included documents.

### Developing the search strategy

The initial theory will be refined using secondary data. The need to find relevant data to develop the programme theory will guide searching. Search strategies will be developed and refined with input from the LEG and SG. The search strategies employed to identify relevant literature will be developed iteratively, and re-visited at predetermined milestones, using different permutations and additional concepts [19, 20].

The proposed initial sampling frame will be the following:

- Context: people living with SMI, taking antipsychotics, different types of SMI, different living homes/living environments, dual diagnosis including SMI and intellectual disability.
- Intervention: non-pharmacological interventions including exercise and dietary interventions.
- Mechanisms: triggered by the intervention, to be identified from the programme theory.
- Outcomes: weight, metabolic adverse events, quality of life, adherence, burden, economic. Unanticipated or unintended outcomes, and outcomes considered important by LEG and SG.

Based on previous work [12, 21–24] and international guidance [25], sources will include MEDLINE/PubMed, Embase, Scopus, Web of Science (Core Collection), the Cochrane Library, CINAHL, PsycINFO, Sociological Abstracts and Google Scholar. Additional grey literature will be sought by searching ETHOS (British Library Electronic Thesis Online), ProQuest Dissertations and Theses, OpenGrey (System for Information on Grey Literature in Europe), the King’s Fund Library Database, NHS Evidence and the websites of relevant charities, user groups and professional bodies. Established links with professional networks and relevant NHS organisations will be drawn on to identify further grey literature including unpublished service evaluations.

We will subsequently use ‘cluster searching’ techniques to identify additional papers that might add to the conceptual richness and contextual thickness of our data, including ‘sibling’ (i.e. directly linked outputs from a single study) and ‘kinship’ (i.e. associated papers with a shared contextual or conceptual pedigree) papers [20]. We will also conduct forward and backward citation searches, using Google Scholar and Web of Science, to identify further related papers from the wider literature, and approach the LEG and SG for recommendations on potentially relevant documents or additional sources to search.

Searching will continue until sufficient data is found (‘theoretical saturation’) to conclude that the refined programme theory is sufficiently coherent and plausible [19]. If the volume of the literature retrieved proves unmanageable, we will employ a variety of appropriate sampling strategies (e.g. theoretical sampling, maximum variation sampling) to ensure that we have sufficient focussed but relevant data for programme theory development [26].

### Selection, appraisal and data extraction

Documents identified will be screened against inclusion and exclusion criteria:

#### Inclusion

People living with SMI (schizophrenia, schizoaffective disorder, bipolar disorder and all other non-organic psychoses)

Taking antipsychotics (British National Formulary [BNF] 78, Chapter 4; section 3.6).

Informal (family/friends) carers for people with SMI

Practitioners supporting people with SMI

#### Exclusion

People without SMI

Medication other than antipsychotics.

Children < 18 years old

Selection and appraisal is a two-step process:

1. Potentially relevant documents will initially be screened by title, abstract and keywords by a member of the research team. A 10% random sample will be checked by CD (any disagreements on the boundaries will be resolved with the input of GW).
2. The full texts of this set of documents will be obtained and screened by a member of the research team. They will read the full text of all the documents that have been included after screening based on title and abstract. Documents will be selected for inclusion when they contain data that is relevant to the realist analysis i.e. could inform some aspect of the programme theory. A 10% random sample will be checked by CD (any disagreements on the boundaries will be resolved with the input of GW).

Full texts of the included papers will be uploaded into NVivo qualitative data analysis software. Relevant sections of texts, which have been interpreted as relating to contexts, mechanisms and their relationships to outcomes, will be coded in NVivo. This coding will be inductive (codes created to categorise data reported in included studies), deductive (codes created in advance of data extraction and analysis as informed by the initial programme theory) and retroductive (codes created based on an interpretation of data to infer what the hidden causal forces might be for outcomes). Each new element of data will be used to refine the theory if appropriate, and as the theory is refined, included studies will be re-scrutinised to search for data relevant to the revised theory that may have been missed initially.

The characteristics of the documents will be extracted separately into an Excel spreadsheet, including bibliographic information and details about study design, population and setting.

### Primary data collection

Realist interviews will be conducted to gather additional data to support, refute or refine the programme theory developed from the literature review stream [12]. Realist interviews with service users, informal carers and practitioners will explore our understanding of the developing programme theory [12]. Realist interviews are a sub-type of qualitative interview where the researcher does a ‘show and tell’ with the participant [24]. The interviews will be semi-structured; an interview guide will be developed. The participant is eased in and then questioned in the most neutral way possible about aspects of the programme theory.

Interviews and/or focus groups with service users, practitioners and informal carers will be recorded and transcribed verbatim using professional transcribers. The interviews will be conducted either face-to-face, or if required, for example, due to COVID restrictions or convenience for participants, using video-conferencing e.g. MS Teams.

Up to 30 service users and informal carers, and 20 practitioners or until data saturation is reached will be interviewed. This sample size is additionally informed by our experience in MEMORABLE [12, 27]. The sampling strategy will be informed by the literature review and will be directed by the need to find data relevant to explore and refine the programme theory. Participants will be purposively sampled to ensure representation from key groups including seldom heard populations. Three key groups will be interviewed.

First, people with SMI to allow the targeted exploration of the programme theory with participants with lived experience. Participants will be purposively sampled to ensure diversity in potentially conceptually relevant characteristics including, for example, ethnicity, gender, diagnosis and age.

Second, practitioners (including Community Psychiatric Nurses [CPNs], psychiatrists, GPs, pharmacists, pharmacy technicians, recovery workers, social workers and occupational therapists) who support people with mental health problems will be interviewed. Participants will be purposively sampled to ensure diversity in potentially conceptually relevant characteristics including, for example, locality (rural vs. urban), and the index of deprivation in the area that they work.

Finally, informal (family) carers identified via third sector organisations and/or via service users will be interviewed. Informal carers will either be interviewed with the service user (as part of the dyad) or alone if not recruited as part of a dyad.

Provisionally the interviews and focus groups will explore our programme theory and then enable us to refine our programme theory. Within a realist interview, participants are asked to provide their interpretations and perceptions of the programme theory. Questioning starts with an unfocussed discussion about the programme theory and then gradually ‘drills’ down into different aspects of the programme theory.

NVivo software will be used to organise and understand the qualitative data. Data analysis will take place after each interview and a use realist logic of analysis. The coding process will be similar to that outlined with the secondary data and be inductive, deductive and retroductive.

All interviews will be recorded and transcribed. For quality control, transcript summaries will be shared with participants and feedback elicited as to their veracity. One member of the team will initially conduct data analysis and coding; a second member of the team (CD) will code 10% of interviews to check for consistency in coding, with a third (IM or GW) resolving any disagreements.

The project team will regularly meet up to discuss the analyses of the interviews; findings will also be presented to the LEG and SG. Through discussion and disputation with members of both groups and the project team inferences will be made about how the programme theory should be further refined.

### Data analysis, synthesis and dissemination

Data analysis will use a realist logic of analysis to make sense of the initial programme theory. Data for analysis will be drawn from following sources: (i) documents that have been included in the realist review after screening against inclusion and exclusion criteria and (ii) transcripts of the primary data collected from the interviews and focus groups. We will use the data from both strands to develop ‘Context Mechanism Outcome Configurations’ (CMOCs). These configurations explain how the context (the situations around a person) affect mechanisms that cause outcomes (intended or not).

As part of our process of analysis, a series of questions about the relevance and rigour of content within data sources will be asked [28]:

- Relevance: Are sections of text within this document or transcript relevant to programme theory development?
- Rigour (judgements about trustworthiness): Are these data sufficiently trustworthy to warrant making changes to the programme theory?
- Interpretation of meaning: if relevant and trustworthy, do the contents provide data that may be interpreted as functioning as context, mechanism or outcome?
- Interpretations and judgements about CMOCs: what is the CMOC (partial or complete) for the data that has been interpreted as functioning as context, mechanism or outcome?
- Interpretations and judgements about programme theory: how does this particular (full or partial) CMOC relate to the programme theory? Within this same document or transcript, are there data which inform how the CMOC relates to the programme theory?

Data to inform our interpretation of the relationships between contexts, mechanisms and outcomes will be sought across documents and transcripts, because not all parts of the configurations will always be articulated in the same document or transcript. Interpretive cross-case comparison will be used to understand and explain how and why observed outcomes have occurred, for example, by comparing settings where interventions to limit antipsychotic weight gain have been reported as being ‘successful’ against those which have not; from this, we will understand how context has influenced the results. When working through the questions set out above, where appropriate, we will use the following forms of reasoning to make sense of the data: juxtaposition of data, reconciling of data, adjudication of data and consolidation of data.

Ultimately, our analyses will be used to identify which practical intervention strategies we might be able to use to change existing contexts in such a way that ‘key’ mechanisms are triggered to produce desired outcomes. From this, guidance will be developed for service users and practitioners.

Participants interviewed earlier, and LEG and SG members will provide feedback on the veracity of the refined CMOCs, the proposed intervention strategies and guidance including the most appropriate format and dissemination strategies. They will also advise us on the ‘real world’ feasibility of these strategies. Following this feedback, the guidance will be appropriately modified. We will work with key stakeholder organisations to support the dissemination of our findings and put outputs into practice.

## Discussion

Physical health problems including antipsychotic-induced weight gain are a key issue for people living with SMI and their carers. This project aims to develop (1) guidance for service users and informal carers on how to manage and limit weight gain, (2) guidance for service providers on how to optimally deliver weight loss services and (3) a framework for future intervention design. A realist research study aims to understand: ‘What works for whom, in what circumstances, how and why?’ Such an approach is ideally suited to exploring how complex interventions such as weight management interventions involving human decisions and actions, may or may not work, and thus inform practical guidance.

The key criterion for success is to develop relevant guidance for service users and clinicians, and a framework for intervention strategies using a realist approach. To do so, we need to identify and capture the relevant primary and secondary data to enable us to understand what works, for whom, and in what circumstances. RESOLVE has included appropriate support from an information specialist to ensure that the relevant literature, including the grey literature, is identified. Based on our experience from MEMORABLE, we plan to interview sufficient service users, informal carers and practitioners to obtain lived experience of what is likely to work that is not available from the literature.

## Data Availability

Data from the realist review is available from the corresponding author at reasonable request. Data sharing is not applicable for the qualitative data generated from the interviews.

## Abbreviations

BMI: Body mass index
CMOCs: Context, mechanism and outcome configurations
CPNs: Community psychiatric nurses
LEG: Lived experience group
RCT: Randomised controlled trial
SG: Stakeholder group
SMI: Severe mental illness (SMI)
